# Safety of percutaneous tracheostomy in NeuroICU patients with intracranial pressure monitoring

**DOI:** 10.1186/cc13898

**Published:** 2014-05-28

**Authors:** Irene K Louh, William D Freeman

**Affiliations:** 1 Department of Neurology, Mayo Clinic, 4500 San Pablo Road, Cannaday 2 East Neurology, Jacksonville, FL 32224, USA; 2 Department of Pulmonary, Allergy, and Critical Care Medicine, Columbia University, 622 West 168 Street, PH 8 East, Rm 101, New York, NY 10032, USA

## 

We read with great interest the article by Scales and Cutherbertson on percutaneous dilatational tracheostomy in a previous issue of *Critical Care*[1]. In particular, we appreciated their last comment: ‘Finally, we believe that some patient populations - for example, those with chronic respiratory conditions or underlying neurological injury - may have risk-benefit profiles that differ from general ICU patients, and this should be further explored’. We wholeheartedly agree with their statement about neurosciences intensive care unit (NeuroICU) patients in particular because patients with traumatic brain injury or poor-grade subarachnoid hemorrhage can develop global cerebral edema (GCE), which may peak within the first few days and can last up to 2 weeks - refractory intracranial pressure (ICP) - without advanced intervention or decompressive hemicraniectomy or both. We have found that GCE patients with refractory ICP may be better served by having earlier tracheostomy if the primary brain injury is survivable and the patients have a reasonable prognosis but that it may be wise to wait longer if the prognosis is indeterminate, as the authors mention the risks versus benefit. Finally, we have studied the ICP surge during the dilatational part of percutaneous tracheostomies in our NeuroICU with indwelling ICP monitors [2]. For the most part, we safely managed ICP with mannitol or hypertonic saline or by opening the ventriculostomy drain to allow ventricular fluid out in order to lower ICP during the procedure. Prior to this study, we found little to no information about the safety of this procedure in this group of patients. Therefore, we agree with the authors that a specific risk-versus-benefit ratio of subsets of ICU patients should be analyzed with regard to this procedure.

## Abbreviations

GCE: Global cerebral edema; ICP: Intracranial pressure; NeuroICU: Neurosciences intensive care unit.

## Competing interests

The authors declare that they have no competing interests.

## References

[B1] Scales DC Cuthbertson BH Percutaneous dilatational tracheostomy: mostly safe, but do benefits outweigh risks? Crit Care 2014 18 117 10.1186/cc13761 25029438 PMC4057159

[B2] Louh I Freeman W Safety and tolerability of percutaneous tracheostomy in neurocritical care patients with poor intracranial compliance Crit Care Med 2012 40 U230

